# Characteristics associated with frequent sexually transmitted infection (STI) testing in a community-based sample of gay, bisexual, and other men who have sex with men (GBMSM), United Kingdom, 2024

**DOI:** 10.1371/journal.pgph.0005351

**Published:** 2026-03-27

**Authors:** Lucy Findlater, George Baldry, Ana K. Harb, Dolores Mullen, Dawn Phillips, Erna Buitendam, Catherine M. Lowndes, David Reid, Catherine H. Mercer, John Saunders, Dana Ogaz, Hamish Mohammed

**Affiliations:** 1 United Kingdom Field Epidemiology Training Programme, United Kingdom Health Security Agency, London, United Kingdom; 2 Division of Blood Safety, Hepatitis, STIs and HIV, United Kingdom Health Security Agency, London, United Kingdom; 3 Institute for Global Health, University College London, London, United Kingdom; 4 National Institute for Health and Care Research Health Protection Research Unit in Blood Borne and Sexually Transmitted Infections at University College London in partnership with the United Kingdom Health Security Agency, London, United Kingdom; New York University, UNITED STATES OF AMERICA

## Abstract

In the UK, gay, bisexual, and other men who have sex with men (GBMSM) at risk of sexually transmitted infections (STIs) are recommended quarterly testing, but it is not known how many are following this recommendation. We described prevalence and correlates of frequent STI testing amongst GBMSM. We analysed data from the community-based Reducing Inequalities in Sexual Health (RiiSH) online survey of GBMSM, with recruitment in November-December 2024 via social media and dating apps. Participants were UK residents aged ≥16 years reporting sex with a man in the previous year. We described frequency of STI testing amongst individuals recommended quarterly testing, using proxies for national guidelines for quarterly testing eligibility (over the past three months: new male sexual partner, condomless anal sex, ≥ 10 male partners, or chemsex). We explored factors associated with frequent testing (≥4 tests/past year) using univariate and multivariable logistic regression, adjusting for country of birth and residence, ethnicity, employment, and education. Among 2758 participants (median age 45 years, 88% white), we estimated 2366 (86%) would be recommended quarterly STI testing. Among 2342 with testing information, 562 individuals (24%) met or exceeded this recommendation (≥4 tests), 1107 (47%) had 1–3 tests, and 673 (29%) none. Factors associated with frequent testing were: reporting using HIV-PrEP in the past year (adjusted odds ratio 7.66 (95% confidence intervals 5.77-10.30)), STI diagnosis in the past three months (1.96 (1.45-2.64)), and younger age (1.50 (1.04-2.16), 16–29 years vs ≥ 45 years). Straight/bisexual orientation was associated with less frequent testing than gay/homosexual (0.71 (0.52-0.96)). Overall, data from a large UK community survey suggest only 1 in 4 GBMSM who are recommended quarterly STI testing meet this recommendation, and testing levels vary by HIV-PrEP use, STI history, age, and sexual orientation. These findings provide baseline data for consideration in any updates to STI testing guidelines.

## Introduction

Gay, bisexual, and other men who have sex with men (GBMSM) have disproportionately high rates of diagnosed sexually transmitted infections (STIs) [1–3]. STI guidelines in the UK and other high income countries recommend that all GBMSM test for STIs and HIV annually and those at increased risk of these infections test quarterly (three-monthly) [4–8]. Additionally, pre-exposure prophylaxis for HIV (PrEP) has been routinely available at sexual health services in the UK since autumn 2020 and quarterly STI and HIV screening is recommended [9]. In the UK, STI testing is free, provided through in-person clinics or online postal self-sampling kits, and includes ‘triple site’ (pharyngeal, rectal, and urethral) screening for chlamydia and gonorrhoea, aiming to detect and treat asymptomatic infections which could facilitate further transmission [10–15].

While guidelines recommend frequent testing for GBMSM, there is uncertainty as to whether they optimally balance benefits and harms, such as the risk of increasing antimicrobial resistance and treatment of asymptomatic infections which may self-resolve [16–21]. Additionally, there is limited published data on the actual STI testing frequency amongst GBMSM; recommended testing frequencies may not be achieved in practice and may vary amongst different subgroups [22,23]. Given evolving sexual health policies, including routine provision of PrEP, antibiotic resistance concerns, and service pressures, contemporary data on STI testing frequency and correlates of frequent testing are needed to inform testing guidelines and resource allocation.

Using data from the 2024 ‘Reducing Inequalities in Sexual Health’ (RiiSH) community survey, this paper examines the frequency of STI testing amongst GBMSM, assesses the extent to which it aligns with current guidelines, and identifies demographic, behavioural, and service-use factors associated with frequent testing.

## Methods

### Ethics statement

Ethical approval for this study was granted by the UKHSA Research and Ethics Governance Group (REGG; ref: R&D 524) and all methods were performed in accordance with guidelines and regulations set by that group. The study used a self-completed online questionnaire that did not collect any direct identifiers such as name or date of birth. Written informed consent was obtained electronically from participants prior to participation. Young people aged 16–17 years were eligible to participate in this survey as this group represent a key population at risk of STI acquisition in the UK and are important to include in this research. Parental consent of participants aged 18 or younger was not sought. Ethical guidelines produced by the British Psychological Society and General Medical Council suggest consent from parents should be sought for those under age 16 and those aged 16 and over may be presumed to be able to reach informed consent if the information on the study, and the way that data is collected, stored, and used is clear. This information was included for all potential participants at the beginning of the survey.

### Study population

Self-identifying men (cisgender or transgender), transgender women, or gender-diverse people who were assigned male at birth (AMAB), aged at last 16 years, resident in the UK, and reporting sex in the past 12 months with a man (cisgender or transgender) or non-binary person AMAB, participating in the 2024 round of the RiiSH survey.

### Study design

Secondary analysis of community-based, cross-sectional, online survey data.

### Data collection

Data collection methods for the RiiSH surveys have been described previously [24–30]. Participants were those who responded to an advert on social networking sites (Facebook, Instagram) or on geospatial dating applications (Grindr, Scruff, Jack’d, and Recon) between 19 November 2024 and 9 December 2024. Users who clicked on the advert were taken to the online survey where they were asked initial questions about eligibility. Eligible individuals were UK residents, aged 16 years or older, men (cisgender or transgender), transgender women, or gender-diverse and assigned male at birth, who reported sex in the past 12 months with a man (cisgender or transgender) or non-binary person assigned male at birth. Online consent for study participation was obtained from eligible individuals and no financial incentive was offered. Ineligible individuals existed the survey. The study used a self-completed, online questionnaire administered via Snap Surveys platform (www.snapsurveys.com), taking approximately 15 minutes to complete. Question topics included HIV/STI testing, vaccination, PrEP use, STI symptoms, use of sexual health services, sexual relationships and behaviour, and drug and alcohol use (S1 Table).

Details on the piloting of the survey are described elsewhere [22]. Briefly, cognitive interviews were conducted with GBMSM to gauge survey acceptability, comprehension of questions, and time taken to complete survey. Subsequent rounds of the survey were extensively piloted by the study team to ensure correct routing and question content. Programmed routing and skip logic was implemented to ensure respondents were only presented with questions relevant to their prior answers, reducing respondent burden and minimising inconsistent responses and item non-response. Soft prompts asked respondents about the plausibility of some answers, with the ability to go back. Survey data were analysed to identify and assess potential duplicates for removal when cleaning. Start and end times of survey completion and response patterns were reviewed with no indication of suspicious coding or duplication.

### Data analysis

Data analysis was conducted using R version 4.2.1. Data were accessed for the purpose of this study on 17 February 2025. Authors were not able to identify participants during or after data collection because participants did not provide information which could identify them in the survey.

In this analysis, ‘testing’ included people testing for STIs if they presented with symptoms, after partner notification, or being screened asymptomatically. We assessed whether a participant would be recommended quarterly testing using UK national guidelines for the sexual health care of GBMSM [7,8]. These guidelines recommend that quarterly STI and HIV screening should be offered to people with any of the following risk factors for STIs and bloodborne viruses: multiple or anonymous sexual partners since last tested, condomless anal sex (CAS) with partners of unknown or serodiscordant HIV status over the past year, any unprotected sexual contact with a new partner since last tested, or drug use during sex (chemsex) over the past six months. We used the following variables to approximate these criteria: any new male sexual partner, CAS, at least 10 male sexual partners, or chemsex, in the past three months (Table 1). The threshold of at least 10 partners reflects the long-tailed distribution of testing in the dataset with a high number of cases reporting at least 10 partners (S1 Fig). We considered using a lower threshold of at least 5 partners and performed a sensitivity analysis using this threshold, but we did not include this as it led to almost no difference (an increase of 4 individuals from 2366 to 2370) in the sample of participants we would consider eligible for quarterly testing. This is because most individuals with 5–9 partners met one of the other eligibility criteria (any new male sexual partner, CAS, or chemsex).

**Table 1:** Criteria for quarterly STI and HIV testing from UK national guidelines and proxy variables used.

Criteria for quarterly STI and HIV testing from national guidelines	Proxy criteria
Multiple or anonymous sexual partners	At least 10 male sexual partners in the past three months (at least 5 partners was considered as a sensitivity analysis)
Condomless anal sex with partners of unknown or serodiscordant HIV status over the past year	Condomless anal sex in the past three months
Any unprotected sexual contact with a new partner since last tested	Any new male sexual partner in the past three months
Drug use during sex (chemsex) over the past six months	Drug use during sex (chemsex) in the past three months

Table comparing guideline criteria for recommending quarterly STI and HIV testing from UK national guidelines with the proxy criteria used for analysis [7,8].

Amongst participants who would be eligible for the quarterly testing recommendation, we described the self-reported frequency of STI testing, defined as number of tests taken in the past year. A variable as a proxy for quarterly STI screening (yes or no) was created, defined as testing at least four times in the past year.

We described where the participant had ever undergone STI testing (e.g., sexual health services, self-testing, hospital, GP, community testing services), and if they had ever received an HIV test, if this was within the last year, and where the last HIV test was received (e.g., sexual health services, self-testing, hospital, GP, community testing services). We also calculated the frequency of STI symptoms and diagnoses amongst this group in the past three months, as well as reasons for visiting sexual health services. Confidence intervals for proportions were estimated using the Wilson score method.

To examine the association between frequent STI testing and sociodemographic and behavioural characteristics, univariable and multivariable logistic regression models were used to produce unadjusted (uOR) and adjusted odds ratios (aOR). We used complete case analysis whereby only participants with complete data for each variable in the model were included. Variable inclusion in the model was primarily informed by existing literature on STI testing behaviours and subject matter knowledge. Variables with established or plausible associations with STI testing frequency, including age, ethnicity, STI history, and sexual orientation, were chosen *a priori* for model inclusion irrespective of statistical significance [25,27]. Univariable models were used secondarily to avoid exclusion of potentially relevant variables, using a p value ≤ 0.2 in univariable analysis for inclusion in multivariable analysis. It is worth noting that the odds ratios calculated from this cross-sectional study reflect associations and should not be interpreted as causal. Model calibration was assessed using the Hosmer-Lemeshow goodness-of-fit test, whereby a higher p value for the test result (p > 0.05) indicates adequate model fit and that the model’s predicted probabilities correspond well with observed outcomes. Multicollinearity among exposure variables was assessed using adjusted generalised Variance Inflation Factor (GVIF), where GVIF values above 5 could indicate problematic multicollinearity.

The group of participants who did not report testing quarterly included people who reported not having had an STI test in the past year or ever. This meant that our analysis could be identifying factors associated with having any testing at all or having ever engaged with sexual health services, as opposed to factors associated with frequent testing. To explore this, we conducted a sensitivity analysis comparing people who tested at least four times in the past year to those who tested between one and three times in the past year. We used the same approach as described in the previous paragraph to build a multivariable logistic regression model and assess which variables were associated with testing at least four times in the past year as opposed to testing one, two, or three times.

## Results

### Sample characteristics

**Fig 1:**
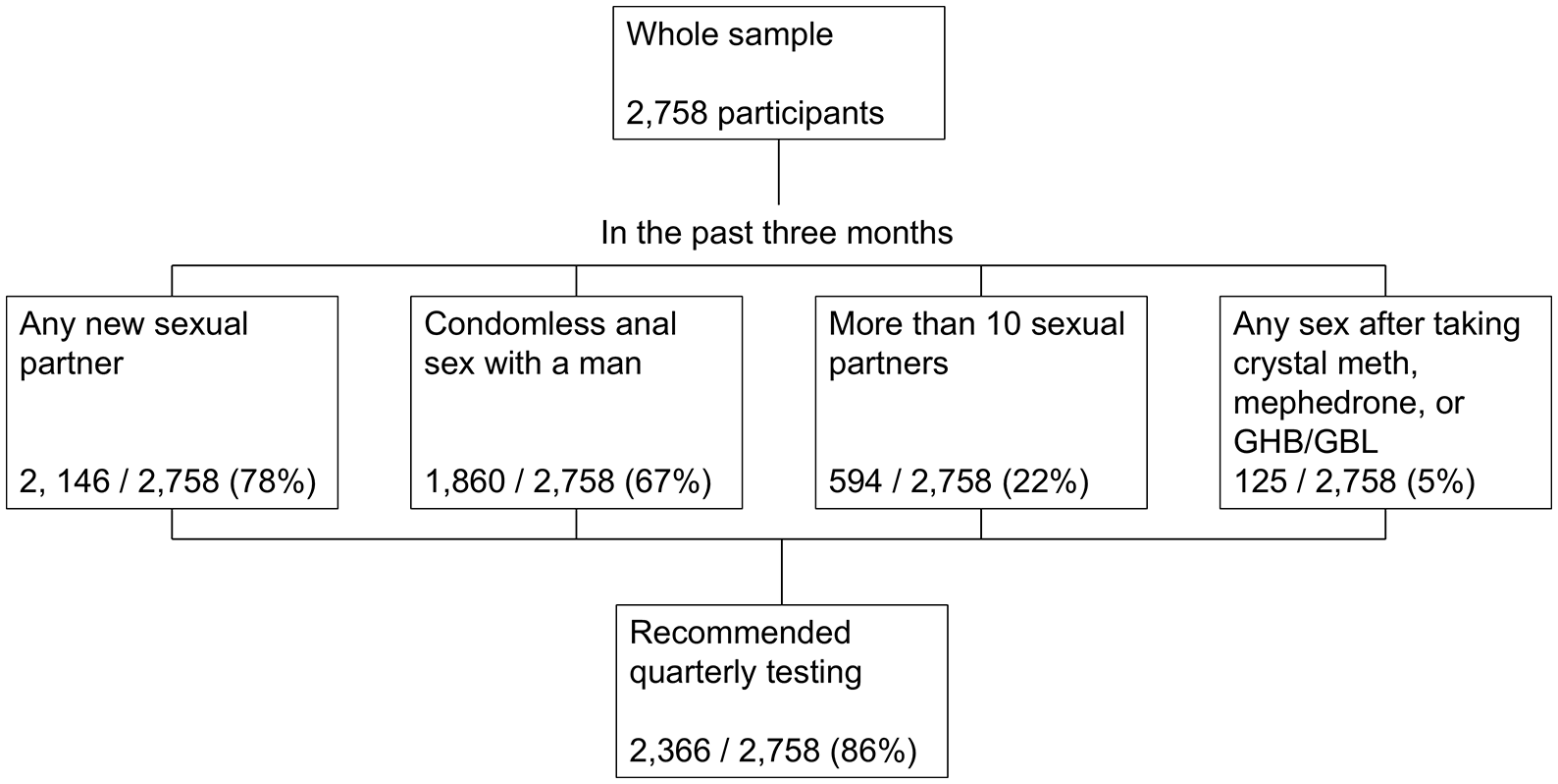
Flowchart describing participants who would be recommended quarterly testing from the RiiSH sample, 2024. Number and proportion of participants in the Reducing Inequalities in Sexual Health (RiiSH) survey 2024 who we estimated would be recommended quarterly testing based on reporting at least one risk factor for STIs over a three-month lookback period (November 2024 looking back to August 2024). The categories are not mutually exclusive and participants could report more than one risk factor.

Overall, 2758 participants were included in RiiSH 2024, with a median age of 45 years (Table 2). Most participants were of white ethnicity (88%), followed by Asian (5%), Mixed or any ‘other’ ethnicity (5%), and Black (2%). Most participants identified as gay or homosexual (80%), with the remaining 20% identifying as bisexual, straight/heterosexual, or other. The majority identified as cisgender male (95%), with the remaining 5% identifying as another gender. We estimated that 2366/ 2758 (85.8%, 95% confidence interval (CI): 84.4% - 87.1%) would be recommended quarterly STI testing based on reporting any new sexual partner (2146), condomless anal sex with a man (1860), more than 10 sexual partners (594), or chemsex (125) (Fig 1). Of these 2366, 58 (2%) participants reported all four indicators, 528 (22%) reported three, 1129 (48%) reported two, and 651 (28%) reported one.

**Table 2:** Characteristics of RiiSH participants overall and of RiiSH participants who would be recommended quarterly STI testing, 2024.

Characteristic	Whole sample (N = 2758)	Recommended quarterly testing (N = 2366)	Not recommended quarterly testing (N = 392)
Age (years)	Median 45 (IQR 36 – 55)	Median 45 (IQR 36 – 55)	Median 45 (IQR 35 – 56)
Age group (years)			
16-29	337 (12%)	275 (12%)	62 (16%)
30-44	1,010 (37%)	878 (37%)	132 (34%)
45 and over	1,411 (51%)	1,213 (51%)	198 (51%)
Gender			
Cisgender male	2,631 (95%)	2,272 (96%)	359 (92%)
Other gender	127 (4.6%)	94 (4.0%)	33 (8.4%)
Sexual orientation			
Gay/homosexual	2,207 (80%)	1,908 (81%)	299 (76%)
Bisexual, straight/heterosexual, or other	551 (20%)	458 (19%)	93 (24%)
Country			
England	2,404 (87%)	2,064 (87%)	340 (87%)
Scotland	194 (7.0%)	165 (7.0%)	29 (7.4%)
Wales	100 (3.6%)	82 (3.5%)	18 (4.6%)
Northern Ireland	60 (2.2%)	55 (2.3%)	5 (1.3%)
Region within England			
East Midlands	168 (6.1%)	134 (5.7%)	34 (8.7%)
East of England	206 (7.5%)	173 (7.3%)	33 (8.4%)
London	748 (27%)	663 (28%)	85 (22%)
North East	89 (3.2%)	74 (3.1%)	15 (3.8%)
North West	316 (11%)	276 (12%)	40 (10%)
South East	323 (12%)	285 (12%)	38 (9.7%)
South West	194 (7.0%)	165 (7.0%)	29 (7.4%)
West Midlands	162 (5.9%)	125 (5.3%)	37 (9.4%)
Yorkshire and Humber	189 (6.9%)	161 (6.8%)	28 (7.1%)
Unknown	9 (0.3%)	8 (0.3%)	1 (0.3%)
Ethnicity			
White	2,435 (88%)	2,094 (89%)	341 (87%)
Asian	141 (5.1%)	119 (5.0%)	22 (5.6%)
Mixed or ‘other’	125 (4.5%)	107 (4.5%)	18 (4.6%)
Black	54 (2.0%)	43 (1.8%)	11 (2.8%)
Unknown	3 (0.1%)	3 (0.1%)	0 (0%)
Education			
Degree or higher	1,628 (59%)	1,443 (61%)	185 (47%)
Higher education below degree	310 (11%)	271 (11%)	39 (9.9%)
A-levels, Scottish Highers, equivalent	292 (11%)	238 (10%)	54 (14%)
GCSEs, O-Levels, National 5, equivalent	380 (14%)	305 (13%)	75 (19%)
No qualifications	70 (2.5%)	46 (1.9%)	24 (6.1%)
Other	78 (2.8%)	63 (2.7%)	15 (3.8%)
Employed	2,139 (78%)	1,863 (79%)	276 (70%)
Used PrEP in last year			
Yes	1,248 (45%)	1,172 (50%)	76 (19%)
No	1,234 (45%)	957 (40%)	277 (71%)
N/A	276 (10%)	237 (10%)	39 (9.9%)
HIV status			
Negative/unknown	2,458 (89%)	2,107 (89%)	351 (90%)
PLWHIV (tested positive)	300 (11%)	259 (11%)	41 (10%)
STI positive since August 2023	284 (10%)	279 (12%)	5 (1.3%)
Number of sexual partners since August 2024			
No sex/only virtual	247 (9.0%)	1 (<0.1%)	246 (63%)
1	309 (11%)	216 (9.1%)	93 (24%)
2-4	844 (31%)	799 (34%)	45 (11%)
5-9	607 (22%)	600 (25%)	7 (1.8%)
10 or more	751 (27%)	750 (32%)	1 (0.3%)
Any new partner since August 2024			
Had multiple partners and more than one partner was new	1,784 (65%)	1784 (75%)	0 (0%)
Had multiple partners and one was new	250 (9.1%)	250 (11%)	0 (0%)
Had only one partner who was also new	112 (4.1%)	112 (4.7%)	0 (0%)
No new partners	612 (22%)	220 (9.3%)	392 (100%)

Characteristics of participants in the Reducing Inequalities in Sexual Health (RiiSH) survey 2024, stratified based on whether they would be recommended quarterly testing for sexually transmitted infections (STIs) according to proxy eligibility criteria (any new male sexual partner, condomless anal sex, at least 10 male sexual partners, or chemsex, in the past three months). PrEP: pre-exposure prophylaxis for HIV. PLWHIV: person living with HIV.

### STI testing frequency

**Fig 2:**
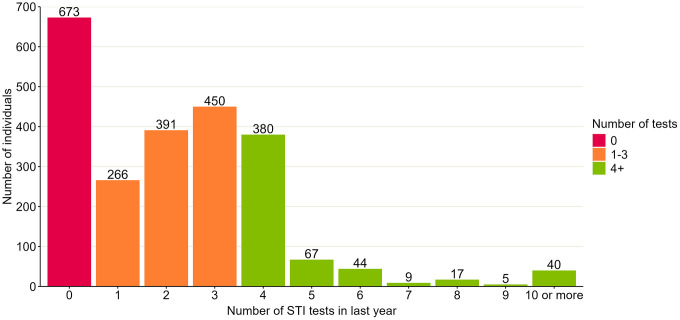
Number of STI tests reported by those RiiSH 2024 survey participants estimated to be recommended to test quarterly. Graph includes 2342/2366 (99%) participants in the Reducing Inequalities in Sexual Health (RiiSH) survey 2024 who had information available on number of STI tests in the past year. Of 24 missing this information, 9 did not know if they had ever STI tested, 11 reported testing in the past year but did not remember how many times, and 4 preferred not to say.

Of 2366 individuals who would be recommended quarterly testing, where STI testing frequency was available (2342), we found that 562 individuals (24.0%, 95% CI: 22.3% - 25.8%) reported testing at least four times in the past year, 1107 (47.3%, 95% CI: 45.3% - 49.3%) tested between one and three times, and 673 (28.7%, 95% CI: 26.9% - 30.6%) did not test in the past year (including 215 (9.2%, 95% CI: 8.1% - 10.4%) who reported that they had never tested for STIs) (Fig 2). There were 40 (1.7%. 95% CI: 1.3% - 2.3%) individuals who reported testing at least ten times in the past year. Testing frequency was estimated from the annual reported number of tests; as test dates were not collected in the survey, we could not explore the length of time between successive tests.

### STI and HIV testing experiences

Amongst participants who would be recommended quarterly testing, most reported that they had tested for STIs at least once ever (2142, 91%), and most within the last year (1684, 71%) (Table 3). Attending sexual health services was the most common route that participants had used for STI testing (1666, 70%), followed by using a free online self-sampling kit (932, 39%). Similarly, the majority of participants reported ever having tested for HIV (2231, 96%), with 239 having tested positive (11%). Most participants reported having had an HIV test less than one year ago (1725, 73%). Sexual health services (1400, 59%) and free online self-sampling kits (586, 25%) were how most people had received their last HIV test.

**Table 3:** STI and HIV testing experiences reported by the whole sample and those recommended quarterly testing, RiiSH survey, 2024.

Testing experience	Whole sample (N = 2758)	Recommended quarterly testing (N = 2366)	Not recommended quarterly testing (N = 392)
Have they ever STI tested			
Yes	2435 (88%)	2142 (91%)	293 (75%)
No	304 (11%)	215 (9.1%)	89 (23%)
Don’t know	19 (0.7%)	9 (0.4%)	10 (2.6%)
When did they last STI test			
Less than one year ago	1834 (66%)	1684 (71%)	150 (38%)
One to two years ago	268 (9.7%)	212 (9.0%)	56 (14%)
More than two years ago	333 (12%)	246 (10%)	87 (22%)
Not known to have tested	304 (11%)	215 (9.1%)	89 (23%)
Don’t know	19 (0.7%)	9 (0.4%)	10 (2.6%)
Where have they ever had STI testing (multiple responses possible)			
Sexual health services	1863 (68%)	1666 (70%)	197 (50%)
Free online self-sampling kit	1020 (37%)	932 (39%)	88 (22%)
Self-testing kit	95 (3.4%)	81 (3.4%)	14 (3.6%)
HIV clinic	83 (3.0%)	68 (2.9%)	15 (3.8%)
GP practice	62 (2.2%)	51 (2.2%)	11 (2.8%)
Community HIV testing service	43 (1.6%)	32 (1.4%)	11 (2.8%)
Somewhere else	25 (0.9%)	23 (1.0%)	2 (0.5%)
Private medical practice	25 (0.9%)	23 (1.0%)	2 (0.5%)
Private online self-sampling service	32 (1.2%)	22 (0.9%)	10 (2.6%)
Mobile medical unit	11 (0.4%)	8 (0.3%)	3 (0.8%)
Have they ever received HIV testing			
Last test was negative	2256 (82%)	1972 (83%)	284 (72%)
Tested positive	300 (11%)	259 (11%)	41 (10%)
Never received an HIV test result	202 (7.3%)	135 (5.7%)	67 (17%)
When was their last HIV test			
Less than 1 year ago	1923 (70%)	1725 (73%)	198 (51%)
1–2 years ago	261 (9.5%)	204 (8.6%)	57 (15%)
More than 2 years ago	372 (13%)	302 (13%)	70 (18%)
Never received HIV test result	202 (7.3%)	135 (5.7%)	67 (17%)
Where was their last HIV test			
Sexual health services	1565 (57%)	1400 (59%)	165 (42%)
Free online self-sampling kit	680 (25%)	586 (25%)	94 (24%)
Never received HIV test result	202 (7.3%)	135 (5.7%)	67 (17%)
Self-testing kit	102 (3.7%)	83 (3.5%)	19 (4.8%)
Hospital outpatient	52 (1.9%)	43 (1.8%)	9 (2.3%)
GP	49 (1.8%)	33 (1.4%)	16 (4.1%)
Community HIV testing service	32 (1.2%)	25 (1.1%)	7 (1.8%)
Somewhere else	18 (0.7%)	14 (0.6%)	4 (1.0%)
Private practice	14 (0.5%)	13 (0.5%)	1 (0.3%)
Hospital inpatient	16 (0.6%)	13 (0.5%)	3 (0.8%)
Private online self-sampling	12 (0.4%)	9 (0.4%)	3 (0.8%)
Blood donation	11 (0.4%)	9 (0.4%)	2 (0.5%)
Mobile medical unit	5 (0.2%)	3 (0.1%)	2 (0.5%)
Have they had any sexual health symptoms in the past three months*	487 (18%)	423 (18%)	64 (16%)
If yes, have they visited a healthcare professional about these symptoms			
Yes	266 (55%)	240 (57%)	26 (41%)
No – but have an appointment	16 (3.3%)	12 (2.8%)	4 (1.0%)
No	205 (42%)	171 (40%)	34 (53%)
Tested positive for any STI in past three months	284 (10%)	279 (12%)	5 (1.3%)
Tested positive for a bacterial STI past three months**	271 (9.8%)	266 (11%)	5 (1.3%)

Sexually transmitted infection (STI) and HIV testing experiences reported by participants in the Reducing Inequalities in Sexual Health (RiiSH) survey 2024. *Full list of symptoms is available in S1 Table. **Bacterial STIs were defined as gonorrhoea, chlamydia, or syphilis.

Of those recommended quarterly testing, 423 (18%) reported experiencing any sexual health symptoms in the past three months. Of these, 252/ 423 (60%) reported visiting a healthcare professional about these symptoms or having an appointment booked. Overall, 266/ 2366 (11%) had tested positive for a bacterial STI in the past three months.

### Attendance at sexual health services

As well as questions about STI testing, participants were also asked about attendance at sexual health services, specifically face-to-face appointments. Of 2366 participants recommended quarterly testing, 2083 (88%) had ever visited a sexual health service, 1499 (63%) in the past year, and 1052 (44%) in the past three months (Table 4). The most common reasons for attending a sexual health service were wanting an STI test or general sexual health check-up (57%), to get PrEP (33%), worry about having an STI or HIV without having symptoms (11%), or having symptoms (11%).

**Table 4:** Attendance at sexual health services and reason for attendance, RiiSH survey, 2024.

	Whole sample (N = 2758)	Recommended quarterly testing (N = 2366)	Not recommended quarterly testing (N = 392)
Ever visited a sexual health service (face-to-face)	2362 (86%)	2083 (88%)	279 (71%)
Attended a sexual health service in the past year	1624 (59%)	1499 (63%)	125 (32%)
Attended a sexual health service in the past three months	1117 (41%)	1052 (44%)	65 (17%)
Why did you last attend a sexual health service (multiple responses possible)			
I wanted an STI test or general sexual health check up	1506 (55%)	1338 (57%)	168 (43%)
To get pre-exposure prophylaxis to prevent HIV	838 (30%)	787 (33%)	51 (13%)
I had no symptoms but was worried I might have an STI or HIV	290 (11%)	251 (11%)	39 (9.9%)
I had symptoms	284 (10%)	259 (11%)	25 (6.4%)
Ongoing HIV care and treatment	183 (6.6%)	158 (6.7%)	25 (6.4%)
A sexual partner was diagnosed with an STI	169 (6.1%)	157 (6.6%)	12 (3.1%)
Treatment after a previous positive test	139 (5.0%)	129 (5.5%)	10 (2.6%)
I needed a vaccination	129 (4.7%)	113 (4.8%)	16 (4.1%)
A sexual partner had symptoms	122 (4.4%)	111 (4.7%)	11 (2.8%)
Check-up after a previous positive test	84 (3.0%)	79 (3.3%)	5 (1.3%)
For another reason	77 (2.8%)	59 (2.5%)	18 (4.6%)
To get post-exposure prophylaxis to prevent HIV	72 (2.6%)	67 (2.8%)	5 (1.3%)
I needed condoms	54 (2.0%)	51 (2.2%)	3 (0.8%)
As follow-up to an online test	48 (1.7%)	42 (1.8%)	6 (1.5%)
I couldn’t get an online testing kit	38 (1.4%)	35 (1.5%)	3 (0.8%)
I was told to attend by my GP/doctor or another healthcare professional	21 (0.8%)	20 (0.8%)	1 (0.3%)
Following sexual assault	16 (0.6%)	12 (0.5%)	4 (1.0%)
I needed contraception (other than condoms)	8 (0.3%)	3 (0.1%)	5 (1.3%)
Following domestic violence	5 (0.2%)	3 (0.1%)	2 (0.5%)

Attendance at sexual health services reported by participants in the Reducing Inequalities in Sexual Health (RiiSH) survey 2024. STI: sexually transmitted infection.

### Correlates of frequent STI testing

**Fig 3:**
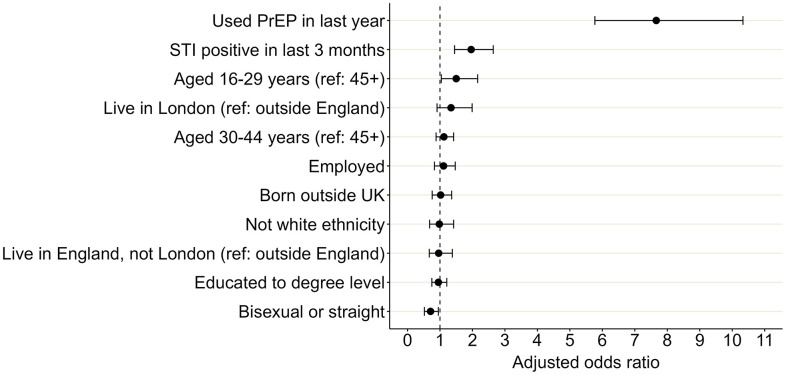
Adjusted odds ratios for factors associated with frequent STI testing (at least four times) compared to those who didn’t test or tested fewer than four times in the past year, RiiSH survey, 2024. Odds ratios showing association of demographic and behavioural factors with frequent testing for sexually transmitted infections (STIs), which was defined as reporting at least four STI tests in the past year, amongst participants in the Reducing Inequalities in Sexual Health (RiiSH) survey 2024. Odds ratios were adjusted for all factors shown in the graph: use of pre-exposure prophylaxis for HIV (PrEP), STI positivity, age, region, employment status, country of birth, ethnicity, level of education, and sexual orientation. Ref = reference group.

Crude and adjusted odds ratios (aORs) are presented in Table 5. In the final multivariable model, the factor most associated with having tested for STIs at least four times in the past year (“frequent testing”) was use of PrEP in the past year (aOR 7.66, 95% CI 5.77 - 10.30) (Fig 3, Table 5). Testing STI positive in the last three months (aOR 1.96, 1.45 - 2.64), or being aged 16–29 years compared to least 45 years (aOR 1.50, 1.04-2.16), were also associated with frequent testing. Participants who identified as straight or bisexual had lower odds of frequent testing compared to those who identified as gay or homosexual (aOR 0.71, 0.52- 0.96). Adjusted generalised Variance Inflation Factor (aGVIF) values for all exposure variables were below 2, suggesting no evidence of problematic multicollinearity (S2 Table). The Hosmer-Lemeshow test indicated adequate goodness of fit test for the final model (χ² = 6.78, degrees of freedom (df) = 8, p = 0.560).

**Table 5:** Crude and adjusted odds ratios for factors associated with frequent STI testing (at least four times) compared to those who did not test or tested fewer than four times in the past year, RiiSH survey, 2024.

	Frequent (N = 562)		Not frequent (N = 1780)		Crude				Adjusted			
Characteristic	%	n/N	%	n/N	Crude OR	Lower CI	Upper CI	P value	Adjusted OR	Lower CI	Upper CI	P value
Used PrEP in last year (ref: no)	87.5%	446/ 510	44.6%	714/ 1600	8.65	6.51	11.62	< 0.001	7.66	5.77	10.30	<0.001
Tested STI positive in the past three months (ref: no)	23.3%	131/ 562	8.1%	144/ 1780	3.45	2.64	4.51	< 0.001	1.96	1.45	2.64	<0.001
Straight/bisexual (ref: gay/homosexual)	13.4%	75/ 562	21.1%	376/ 1780	0.58	0.43	0.76	< 0.001	0.71	0.52	0.96	0.026
Age group (years)												
16–29 years	11.6%	65/ 562	11.5%	204/ 1780	1.15	0.84	1.56	0.375	1.50	1.04	2.16	0.029
30–44 years	42.0%	236/ 562	35.6%	633/ 1780	1.35	1.10	1.65	0.004	1.12	0.88	1.42	0.365
45 + years (ref)	46.4%	261/ 562	53.0%	943/ 1780	1.0	–	–	–	1.0	–	–	–
Place of residence												
England – London	37.6%	210/ 559	25.0%	444/ 1776	1.99	1.44	2.79	< 0.001	1.34	0.91	1.99	0.147
England – outside London	52.2%	292/ 559	61.5%	1092/ 1776	1.13	0.83	1.56	0.463	0.96	0.68	1.38	0.805
Outside England	10.2%	57/ 559	13.5%	240/ 1776	1.0	–	–	–	1.0	–	–	–
Employed (ref: not employed)	81.3%	457/ 562	78.1%	1390/ 1780	1.22	0.96	1.57	0.102	1.11	0.83	1.47	0.492
Educated to degree level (ref: other)	65.7%	369/ 562	59.7%	1063/ 1780	1.29	1.05	1.58	0.012	0.95	0.75	1.21	0.668
Born outside UK (ref: born in UK)	26.7%	150/ 562	20.0%	356/ 1780	1.46	1.16	1.82	0.001	1.02	0.76	1.36	0.891
All other ethnic groups combined (ref: white)	11.9%	67/ 561	10.9%	184/ 1778	1.11	0.81	1.50	0.499	0.98	0.68	1.42	0.929

Table describes characteristics of individuals who tested for sexually transmitted infections (STIs) frequently (at least four times in the past year) compared to those who did not test or tested less than four times, amongst participants in the Reducing Inequalities in Sexual Health (RiiSH) survey 2024. Table shows crude and adjusted odds ratios (ORs) and confidence intervals (CIs) obtained using multivariable logistic regression, describing the association of demographic and behavioural factors with frequent testing. PrEP: pre-exposure prophylaxis for HIV. Ref: reference group. Adjusted odds ratios were adjusted for all variables shown in the table. Model includes 2,101/ 2,366 (89%) observations which had complete information. 24 excluded due to missing STI testing frequency because they did not know if they had ever tested (9) or how many times they tested (11) or preferred not to say (4). 237 excluded due to missing PrEP use in past year because they had previously tested positive for HIV (227) or did not know if they had ever used PrEP (10). 3 excluded due to not providing information on ethnicity. 8 excluded due to missing information on region of residence within England.

### Sensitivity analysis

When we compared those who tested at least four times in the past year with those who had tested at least once but fewer than four times, the same risk factors were identified as in the main analysis (S3 Table). Use of PrEP (aOR 3.99, 2.96-5.45) and having tested positive for an STI (aOR 1.51, 1.13-2.03) were still the factors most strongly associated with frequent testing, but with a lower magnitude of effect than when our reference group included those who hadn’t tested at all in the past year. This suggests that use of PrEP and recent STI history are associated with having at least one test as well as increased frequency of testing. Younger age (16–29 years compared to over 45 years of age) was also associated with more frequent testing (aOR 1.56, 1.06-2.28), whilst identifying as straight or bisexual was associated with lower odds of frequent testing compared to those who identified as gay or homosexual (aOR 0.72, 0.52- 0.98), both of which at a very similar magnitude to the main analysis. This similarity of effect estimates after excluding non-testers suggests that these factors are associated with frequency of testing rather than with testing alone. There was no evidence of problematic multicollinearity from aGIF values and the Hosmer-Lemeshow test indicated adequate goodness of fit test for the final model (χ^2^ = 8.27, degrees of freedom (df) = 8, p = 0.407) (S4 Table).

## Discussion

As different countries reevaluate their asymptomatic STI testing frequency recommendations, it is important to understand how often individuals are currently testing. Our analysis of data from a large, national community survey of GBMSM suggest that whilst the majority would be recommended to test for STIs every three months, only a quarter met this recommendation. Just under half (47%) reported that they had tested between one and three times, and the remainder (29%) had not tested for STIs in the past year. STI testing had usually taken place at sexual health services, followed by free self-sampling kits ordered online. Just under half of participants who would be recommended quarterly testing had attended sexual health services for a face-to-face appointment in the past three months. Factors most strongly associated with frequent STI testing were use of PrEP in the last year, STI diagnosis in the past three months, identifying as gay or homosexual rather than straight or bisexual, and younger age.

Our study indicates that not all GBMSM at the highest risk of STIs are testing as often as recommended in national guidelines. Our findings align with previous research to indicate that testing levels may be lower than expected, with a UK study in 2019 estimating that GBMSM tested an average of 2.1 times per year [31]. In Australia, when national guidelines recommended quarterly testing for sexually active GBMSM not in monogamous relationships, a survey of GBMSM in 2021 found that only 31% had tested for STIs at least three times in the past year [32]. A US study in 2017 estimated that 34% of GBMSM surveyed at community venues had not tested for STIs in the past year [33]. Our analysis of contemporary data is particularly relevant in the current context of uncertainty regarding the benefits and harms of frequent STI testing, including the risk of antimicrobial resistance [15,21,34]. Any move to change testing guidance, in the UK or internationally, should consider that the frequency of STI testing amongst GBMSM is likely to be considerably lower than is currently recommended.

We found that PrEP use was strongly associated with frequent STI testing. This is not surprising given that in the UK, people prescribed PrEP are recommended quarterly HIV/STI screening [35]. An Australian survey in 2021 similarly showed that the proportion of GBMSM who had received STI testing in the past year was much higher amongst those taking PrEP (59%) than those not taking PrEP (8%) [32]. People who use PrEP are self-identifying or clinically assessed as having a higher risk of exposure to HIV (and thus to STIs), and integration of routine STI testing within PrEP delivery appears to facilitate more regular STI testing among these individuals at higher risk. This supports the continued integration of STI screening within PrEP services as a targeted public health strategy. It is also understandable that a recent STI diagnosis would be associated with frequent testing, as at least one test would be required for diagnosis, and possibly further tests of cure. UK guidance goes further to recommend that anyone with a bacterial STI diagnosis in the past year undertakes three-monthly testing [7]. Our findings suggest that these people with greater risk of STIs based on a prior diagnosis are more likely to meet these testing recommendations, highlighting that engagement following STI diagnosis may represent a key opportunity for reinforcing testing and prevention messaging.

Amongst participants at risk of STI infection, GBMSM aged over 45 years appeared to be less likely to undergo frequent STI testing. Limited current evidence is available on the relationship between STI testing frequency and age amongst GBMSM. Studies have suggested that GBMSM of older age groups may be less likely to test for HIV or receive comprehensive HIV/STI counselling, which may also be reflected in reduced STI testing frequency [36,37]. Sexual orientation also appears to be associated with STI testing frequency, with straight or bisexual-identifying GBMSM less likely to test frequently. Another research study in the UK reported that MSM identifying as heterosexual were 30% less likely than those identifying as gay or homosexual to have tested for STIs in the past year [38]. These findings indicate that uniform testing guidance may insufficiently address disparities in testing uptake and highlights the need for tailored interventions to improve engagement among specific subgroups.

Our study utilised a detailed, community-based survey which successfully reached a large number of GBMSM in the UK, many of whom we estimated to have a higher risk of STIs. Our findings give an indication of current STI testing patterns and correlates in this population, providing evidence to feed into decisions on STI testing guidance for GBMSM in the UK. Our study was limited in that, as a cross-sectional survey, it could not be used to explore causality or temporality of behaviours in relation to STI testing. We used proxy definitions for three-monthly testing, describing number of tests in a year rather than timing of tests, which could overestimate quarterly testing if tests are clustered in time rather than spaced evenly throughout the year. However, people are recommended not to test more often than quarterly, to manage service demand and to reduce the risk of false positive results after detection of residual material from a recent infection, and it is unlikely many people would test more than quarterly [7]. Additionally, the proxy definitions we used for whether an individual would be recommended quarterly testing do not perfectly align with the national guidelines which could lead to misclassification bias. This could overestimate eligibility for quarterly testing (for example, including participants reporting condomless anal sex within monogamous relationships) or underestimate eligibility (excluding participants with multiple but fewer than 10 sexual partners). Behaviours were self-reported and could be affected by social desirability bias, which may lead to over-reporting of frequent testing, or recall bias and potentially under-reporting if participants do not accurately remember their testing history. The magnitude of these biases is unknown. Due to small numbers, we also binarised some variables (for example, ethnicity and country of birth) and could not explore further the differences between these groups. Recruitment through online surveys on dating applications could introduce selection bias by preferentially capturing GBMSM with a higher risk of STIs, more connected to online sexual networks, and more willing to engage in sexual health research. However, online rather than in-person recruitment at sexual health clinics may have enabled participation of individuals who engage less with sexual health services [22]. Comparing to GBMSM population demographics, RiiSH participants were more commonly of older age and white ethnicity; therefore, findings may be less reflective of experiences of other ethnic groups or younger GBMSM [39,40].

Overall, we found that only 1 in 4 GBMSM with an estimated greater risk of STIs tested as often as recommended. PrEP use, a recent STI diagnosis, and younger age were associated with testing more frequently, whilst straight or bisexual identification was associated with less frequent testing. Any revision to STI testing guidance should take into account that actual testing frequency in GBMSM is likely to be substantially lower than current testing recommendations, and to vary by demographic and behavioural factors.

## Supporting information

S1 Fig Number of male sexual partners in the previous three months reported by participants in the RiiSH survey, 2024. Graph showing the number of sexual partners in the previous three months reported by participants in the Reducing Inequalities in Sexual Health (RiiSH) survey 2024.
(TIF)

S1 Table Sexual health symptoms listed in RiiSH 2024 questionnaire. Sexual health symptoms asked about in the Reducing Inequalities in Sexual Health (RiiSH) survey 2024. Participants were asked different questions about sexual health symptoms depending on the sex they were assigned at birth, which could be AMAB (assigned male at birth), AFAB (assigned female at birth), AIAB (assigned intersex at birth), or PNS (prefer not to say).
(DOCX)

S2 Table Multicollinearity in model exploring factors associated with frequent STI testing (at least four times) compared to those who didn’t test or tested fewer than four times in the past year, RiiSH survey, 2024 Table presents adjusted Generalised Variance Inflation Factor (aGVIF) values to explore multicollinearity among exposure variables included in the multivariable logistic regression model exploring factors associated with frequent compared to less frequent or no STI testing. Low aGVIF values (less than 5) suggests there is no evidence of problematic multicollinearity in the model.
(DOCX)

S3 Table Crude and adjusted odds ratios for factors associated with frequent STI testing (at least four times) compared to less frequent testing (one, two, or three times) in the past year, RiiSH survey, 2024. Table describes characteristics of individuals who tested for sexually transmitted infections (STIs) frequently (at least four times in the past year) compared to those who tested one, two, or three times, amongst participants in the Reducing Inequalities in Sexual Health (RiiSH) survey 2024. Table shows crude and adjusted odds ratios (ORs) and confidence intervals (CIs) obtained using multivariable logistic regression, describing the association of demographic and behavioural factors with frequent testing. PrEP: pre-exposure prophylaxis for HIV. PLWHIV: Person living with HIV. Ref: reference group. Adjusted odds ratios were adjusted for all variables shown in the table. Model includes 1,669/ 1,693 (99%) observations which had complete information, after we had excluded 673 participants who had not tested in the past year. 24 were excluded due to missing STI testing frequency because they didn’t know if they had ever tested (9) or how many times they tested (11) or preferred not to say (4). 177 excluded due to missing PrEP use in past year because they had previously tested positive for HIV (175) or did not know if they had ever used PrEP (2). 3 excluded due to not providing information on ethnicity. 4 excluded due to missing information on region of residence within England.
(DOCX)

S4 Table Multicollinearity in model exploring factors associated with frequent STI testing (at least four times) compared to less frequent testing (one, two, or three times) in the past year, RiiSH survey, 2024. Table presents adjusted Generalised Variance Inflation Factor (aGVIF) values to explore multicollinearity among exposure variables included in the multivariable logistic regression model exploring factors associated with frequent compared to less frequent STI testing. PLWHIV: Person living with HIV. Low aGVIF values (less than 5) suggests there is no evidence of problematic multicollinearity in the model.
(DOCX)
