# Supplementary figures and images for: Characteristics associated with frequent sexually transmitted infection (STI) testing in a community-based sample of gay, bisexual, and other men who have sex with men (GBMSM), United Kingdom, 2024
Source: PLOS Glob Public Health. 2026 Mar 27;6(3):e0005351. doi: 10.1371/journal.pgph.0005351 (PMC13029752; doi:10.1371/journal.pgph.0005351)

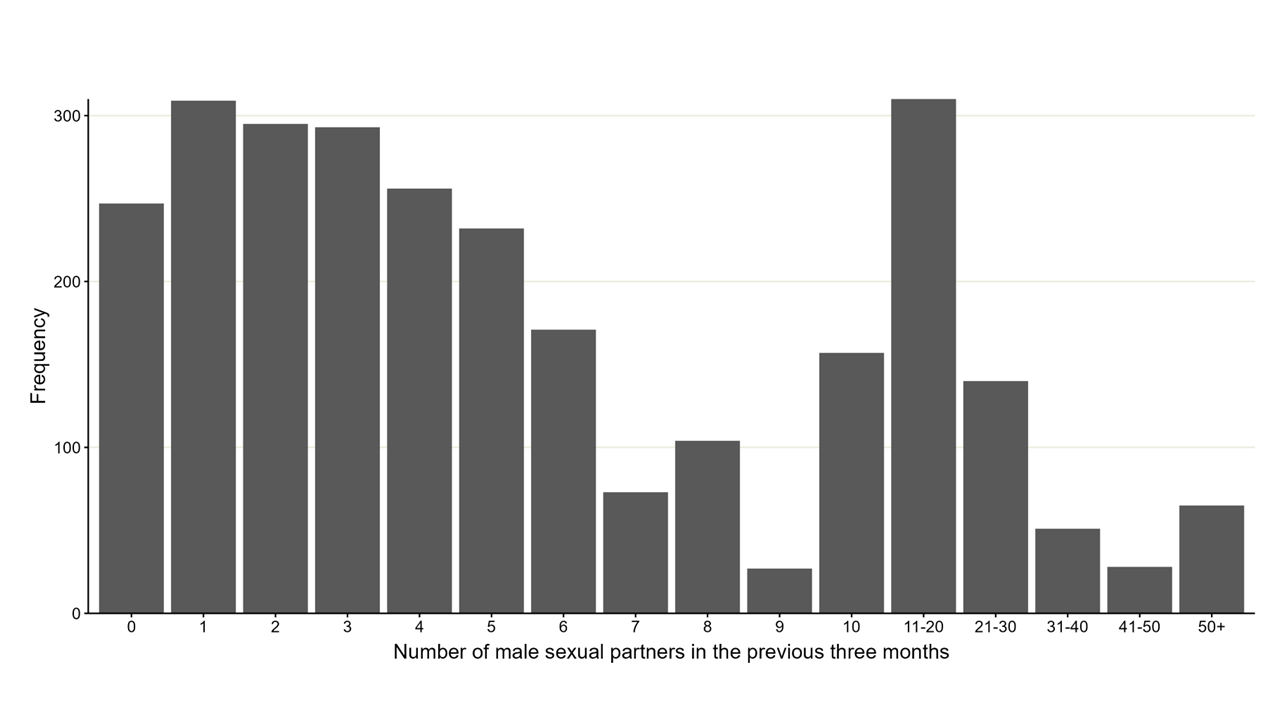

Supplement: S1 Fig — Graph showing the number of sexual partners in the previous three months reported by participants in the Reducing Inequalities in Sexual Health (RiiSH) survey 2024. (TIF) [file pgph.0005351.s001.tif]
