# Supplementary material for: Characteristics associated with frequent sexually transmitted infection (STI) testing in a community-based sample of gay, bisexual, and other men who have sex with men (GBMSM), United Kingdom, 2024
Source: PLOS Glob Public Health. 2026 Mar 27;6(3):e0005351. doi: 10.1371/journal.pgph.0005351 (PMC13029752; doi:10.1371/journal.pgph.0005351)
Supplement: S1 Table — Sexual health symptoms asked about in the Reducing Inequalities in Sexual Health (RiiSH) survey 2024. Participants were asked different questions about sexual health symptoms depending on the sex they were assigned at birth, which could be AMAB (assigned male at birth), AFAB (assigned female at birth), AIAB (assigned intersex at birth), or PNS (prefer not to say). (DOCX) [file pgph.0005351.s002.docx]

**S1 Table: Sexual health symptoms listed in RiiSH 2024 questionnaire**

| Who was asked | Symptom |
| --- | --- |
| All respondents | Pain, burning, or stinging when passing urine |
| All respondents | Passing urine more often than usual |
| All respondents | Pain, bleeding, or discharge from the rectum |
| All respondents | Pain during sex |
| All respondents | Genital wart or lump |
| All respondents | Genital ulcer or sore |
| AMAB | Discharge from the end of the penis |
| AFAB, AIAB, PNS | Discharge from the end of the penis or discharge from the vagina/ front hole |
| AMAB, AIAB | Painful testicles |
| AFAB, AIAB | Abnormal vaginal discharge |
| AFAB, AIAB | Vaginal pain during sex |
| AFAB, AIAB | Abnormal bleeding between periods |
| AFAB, AIAB | Bleeding after sex (not during a period) |
| AFAB, AIAB | Lower abdominal or pelvic pain (not related to periods) |

**S1 Table legend:** Sexual health symptoms asked about in the Reducing Inequalities in Sexual Health (RiiSH) survey 2024. Participants were asked different questions about sexual health symptoms depending on the sex they were assigned at birth, which could be AMAB (assigned male at birth), AFAB (assigned female at birth), AIAB (assigned intersex at birth), or PNS (prefer not to say).
