# Supplementary material for: Characteristics associated with frequent sexually transmitted infection (STI) testing in a community-based sample of gay, bisexual, and other men who have sex with men (GBMSM), United Kingdom, 2024
Source: PLOS Glob Public Health. 2026 Mar 27;6(3):e0005351. doi: 10.1371/journal.pgph.0005351 (PMC13029752; doi:10.1371/journal.pgph.0005351)
Supplement: S3 Table — Table describes characteristics of individuals who tested for sexually transmitted infections (STIs) frequently (at least four times in the past year) compared to those who tested one, two, or three times, amongst participants in the Reducing Inequalities in Sexual Health (RiiSH) survey 2024. Table shows crude and adjusted odds ratios (ORs) and confidence intervals (CIs) obtained using multivariable logistic regression, describing the association of demographic and behavioural factors with frequent testing. PrEP: pre-exposure prophylaxis for HIV. PLWHIV: Person living with HIV. Ref: reference group. Adjusted odds ratios were adjusted for all variables shown in the table. Model includes 1,669/ 1,693 (99%) observations which had complete information, after we had excluded 673 participants who had not tested in the past year. 24 were excluded due to missing STI testing frequency because they didn’t know if they had ever tested (9) or how many times they tested (11) or preferred not to say (4). 177 excluded due to missing PrEP use in past year because they had previously tested positive for HIV (175) or did not know if they had ever used PrEP (2). 3 excluded due to not providing information on ethnicity. 4 excluded due to missing information on region of residence within England. (DOCX) [file pgph.0005351.s004.docx]

**S3 Table: Crude and adjusted odds ratios for factors associated with frequent STI testing (at least four times) compared to less frequent testing (one, two, or three times) in the past year, RiiSH survey, 2024**

|  | Frequent (n = 562) | | Not frequent (n = 1107) | | Crude |  |  |  | Adjusted |  |  |  |
| --- | --- | --- | --- | --- | --- | --- | --- | --- | --- | --- | --- | --- |
| Characteristic | % | n/N | % | n/N | Crude OR | Lower CI | Upper CI | P value | Adjusted OR | Lower CI | Upper CI | P value |
| Used PrEP in last year (ref: no) | 87.5% | 446/ 510 | 62.3% | 612/ 982 | 4.21 | 3.13 | 5.73 | 0.000 | 3.99 | 2.96 | 5.45 | <0.001 |
| Tested STI positive in the past three months (ref: no) | 23.3% | 131/ 562 | 13.0% | 144/ 1107 | 2.03 | 1.55 | 2.66 | 0.000 | 1.51 | 1.13 | 2.03 | 0.006 |
| Age group (years) |  |  |  |  |  |  |  |  |  |  |  |  |
| 16-29 years | 11.6% | 65/ 562 | 9.6% | 106/ 1107 | 1.37 | 0.97 | 1.92 | 0.073 | 1.56 | 1.06 | 2.28 | 0.021 |
| 30-44 years | 42.0% | 236/ 562 | 37.9% | 419/ 1107 | 1.26 | 1.01 | 1.56 | 0.039 | 1.13 | 0.88 | 1.44 | 0.338 |
| 45+ years (ref) | 46.4% | 261/ 562 | 52.6% | 582/ 1107 | 1.0 | - | - | - | 1.0 | - | - | - |
| Straight/bisexual (ref: gay/homosexual) | 13.4% | 75/ 562 | 18.6% | 206/ 1107 | 0.67 | 0.50 | 0.90 | 0.007 | 0.72 | 0.52 | 0.98 | 0.040 |
| Place of residence |  |  |  |  |  |  |  |  |  |  |  |  |
| England – London | 37.6% | 210/ 559 | 29.0% | 321/ 1106 | 1.69 | 1.19 | 2.41 | 0.004 | 1.36 | 0.91 | 2.04 | 0.134 |
| England – outside London | 52.2% | 292/ 559 | 57.7% | 638/ 1106 | 1.18 | 0.85 | 1.66 | 0.333 | 1.03 | 0.72 | 1.50 | 0.872 |
| Outside England | 10.2% | 57/ 559 | 13.3% | 147/ 1106 | 1.0 | - | - | - | 1.0 | - | - | - |
| Born outside UK (ref: born in UK) | 26.7% | 150/ 562 | 22.0% | 244/ 1107 | 1.29 | 1.01 | 1.64 | 0.035 | 1.05 | 0.78 | 1.41 | 0.764 |
| PLWHIV (ref: tested negative/ unknown) | 10.0% | 56/ 562 | 13.0% | 144/ 1107 | 0.74 | 0.52 | 1.04 | 0.070 | 1.15 | 0.37 | 3.00 | 0.793 |
| All other ethnic groups combined (ref: white) | 11.9% | 67/ 561 | 11.8% | 130/ 1105 | 1.02 | 0.73 | 1.41 | 0.915 | 0.96 | 0.65 | 1.40 | 0.826 |

**S3 Table legend:** Table describes characteristics of individuals who tested for sexually transmitted infections (STIs) frequently (at least four times in the past year) compared to those who tested one, two, or three times, amongst participants in the Reducing Inequalities in Sexual Health (RiiSH) survey 2024. Table shows crude and adjusted odds ratios (ORs) and confidence intervals (CIs) obtained using multivariable logistic regression, describing the association of demographic and behavioural factors with frequent testing. PrEP: pre-exposure prophylaxis for HIV. PLWHIV: Person living with HIV. Ref: reference group. Adjusted odds ratios were adjusted for all variables shown in the table. Model includes 1,669/ 1,693 (99%) observations which had complete information, after we had excluded 673 participants who had not tested in the past year. 24 were excluded due to missing STI testing frequency because they didn’t know if they had ever tested (9) or how many times they tested (11) or preferred not to say (4). 177 excluded due to missing PrEP use in past year because they had previously tested positive for HIV (175) or did not know if they had ever used PrEP (2). 3 excluded due to not providing information on ethnicity. 4 excluded due to missing information on region of residence within England.
