# Supplementary material for: Characteristics associated with frequent sexually transmitted infection (STI) testing in a community-based sample of gay, bisexual, and other men who have sex with men (GBMSM), United Kingdom, 2024
Source: PLOS Glob Public Health. 2026 Mar 27;6(3):e0005351. doi: 10.1371/journal.pgph.0005351 (PMC13029752; doi:10.1371/journal.pgph.0005351)
Supplement: S4 Table — Table presents adjusted Generalised Variance Inflation Factor (aGVIF) values to explore multicollinearity among exposure variables included in the multivariable logistic regression model exploring factors associated with frequent compared to less frequent STI testing. PLWHIV: Person living with HIV. Low aGVIF values (less than 5) suggests there is no evidence of problematic multicollinearity in the model. (DOCX) [file pgph.0005351.s005.docx]

**S4 Table:** **Multicollinearity in model exploring factors associated with frequent STI testing (at least four times) compared to less frequent testing (one, two, or three times) in the past year, RiiSH survey, 2024**

| Characteristic | Adjusted generalised Variance Inflation Factor (aGVIF) |
| --- | --- |
| Used PrEP in last year (ref: no) | 1.03 |
| Tested STI positive in the past three months (ref: no) | 1.01 |
| Straight/bisexual (ref: gay/homosexual) | 1.02 |
| Age group (16-29 years, 30-44 years, or ref: 45+ years) | 1.02 |
| Place of residence (England-London, England-outside London, or ref: outside England) | 1.03 |
| Born outside UK (ref: born in UK) | 1.13 |
| PLWHIV (ref: negative/ unknown) | 1.02 |
| All other ethnic groups combined (ref: white) | 1.09 |

**S4 Table legend:** Table presents adjusted Generalised Variance Inflation Factor (aGVIF) values to explore multicollinearity among exposure variables included in the multivariable logistic regression model exploring factors associated with frequent compared to less frequent STI testing. PLWHIV: Person living with HIV. Low aGVIF values (less than 5) suggests there is no evidence of problematic multicollinearity in the model.
